# Society Meetings

**Published:** 1910-03

**Authors:** 


### SOCIETY MEETINGS.

---

### United States Pharmacopeial Convention

The Ninth Decennial Convention for the Revision of the Pharmacopeia of the United States of America, the United States Pharmacopeial Convention, will be held at Washington, D. C., May 10, 1910.

The following list of delegates is offered for publication "without prejudice," it being understood that such publication does not of necessity mean that these delegates will be received and seated by the convention, as their credentials have not as yet been passed upon by the committee on credentials and arrangements.

Attention is invited to a ruling of the committee on credentials and arrangements, that it can "not recognise the right of any one individual to represent more than one organisation or institution.

American Pharmaceutical Association.—Albert B. Lyons, Charles Holzhauer, Lyman F. Kebler; Alternates, Joseph W. England, Thomas F. Main, Leo Eliel.

American Chemical Society.—Geo. D. Rosengarten, L. W. Andrews, Edward Hart; Alternates, Thos. B. Aldrich, John T. Baker, A. B. Lyons.

Medical Department, U. S. Army.—Walter D. McCaw, Carl R. Darnall, Frederick F. Russell.

Bureau of Medicine and Surgery, Navy Department.—H. G. Beyer, G. L. Angeny, P. J. Waldener.

U. S. Public Health and Marine Hospital Service.—John F. Anderson, Reid Hunt, Martin I. Wilbert.

Arkansas Association of Pharmacists.—Frank Schachleiter, John B. Bond, Sr., W. L. Dewoody; Alternate, Jesse D. Hodge.

Colorado State Medical Society.—E. C. Hill, Geo. A. Moleen, W. T. Little; Alternates, O. M. Gilbert, Geo. W. Miel, Sol Ringolsky.

University of Colorado, Medical Department.—Carroll E. Edson, Alvin R. Peebles, Wm. A. Jolley.

Yale Medical School.—Oliver T. Osborne, Herbert E. Smith, Clarence G. Spalding.

Connecticut Pharmaceutical Association.—Charles A. Rapelye, John K. Williams, Chas. W. Whittlesey.

Delaware Pharmaceutical Society.—H. J. Watson, W. F. Dunn, William Poolé; Alternates, Thomas Donaldson, J. O. Bosley, J. T. Challenger.

Medical Society of the District of Columbia.—Murray Galt Motter, John W. Chapell, D. Webster Prentiss.

George Washington University, Department of Medicine.—Sterling Ruffin, B. M. Randolph, Noble P. Barnes.

Georgetown University, School of Medicine.—George M. Kober, G. Lloyd Magruder, W. M. Barton.

George Washington University, National College of Pharmacy.—Henry E. Kalusowski, Samuel L. Hilton, Lewis Flemer; Alternates, Samuel Waggaman, Frank P. Weller, Wymond H. Bradbury.

Illinois State Medical Society.—N. S. Davis, William E. Quine, Henry B. Hemenway; Alternates, Charles E. Chapin, C. W. Lillie.

Northwestern University, Medical School.—Arthur R. Edwards, John H. Long, Alfred N. Richards.

University of Illinois, Medical Department.—Bernard Fantus; Alternate, Edward L. Heintz.

Illinois Pharmaceutical Association.—Wilhelm Bodeman, W. C. Simpson, Herman Fry; Alternates, Andrew Scherer, Gus Lindvall, C. M. Snow.

Literary and Scientific Society of German Apothecaries of the City of New York.—E. C. Goetting, Wm. C. Alpers, F. Hirseman; Alternates, Carl F. Schleussner, Otto Raubenheimer.

Kings County Pharmaceutical Society.—Otto Raubenheimer, Adrian Paradis, Miss K. C. Mahegin; Alternates, J. H. Rohtuss, C. O. Douden, C. E. Heimerzheim.

North Dakota Pharmaceutical Association.—Oscar Hallenberg.

University of Cincinnati, Medical Department.—Julius Eichberg, L. C. Schrickel.

Ohio State Pharmaceutical Association.—Lewis C. Hopp, Theo. D. Wetterstroem, George B. Topping; Alternates, John C. Firmin, Azor Thurston, Eugene Selzer.

Ohio State University, College of Pharmacy.—George B. Kauffman, C. A. Day, William McPherson; Alternates, D. S. White, W. E. Henderson, J. McI. Phillips.

Western Reserve Unversity, Department of Pharmacy.—Harry V. Army, Joseph Feil, William T. Hankey.

University of Oklahoma, School of Pharmacy.—Homer C. Washburn, Edwin DeBarr, John D. McLaren; Alternates, Albert E. Van Vleet, Louis A. Turley, Henry H. Lane.

Medical Society of the State of Pennsylvania.—Adolph Koenig, David Reisman, Henry Beates; Alternates, James B. Walker, Thomas S. Blair.

College of Physicians of Philadelphia.—H. C. Wood, Jr., Solomon Solis-Cohen, James M. Anders; Alternates, Richard A. Cleeman, Judson Daland, L. F. Appleman.

Woman's Medical College of Pennsylvania.—Henry Leffman, Arthur A. Stevens, Frederick P. Henry.

Pennsylvania Pharmaceutical Association.—Lucius L. Walton, M. N. Kline, W. L. Cliffe; Alternates, C. B. Lowe, D. J. Thomas.

Philadelphia College of Pharmacy.—Joseph P. Remington, Samuel P. Sadtler, Henry Kraemer; Alternates, Frank X. Moerk, Charles H. LaWall.

University of Pittsburg, Department of Pharmacy.—Julius A. Kosh, James H. Beal, Louis Sallbach; Alternates, Louis Emanuel, B. E. Pritchard, George W. Kutscher.

Fort Worth University, Medical Department.—R. H. Needham, W. G. Cook, Wm. R. Howard; Alternates, M. E. Gilmore, I. C. Chase, F. G. Landers.

Texas Pharmaceutical Association.—J. C. Buckner, E. G. Eberle.

Medical Society of Virginia.—Roshier W. Miller, F. M. Read, John Staige Davis.

University of Virginia.—Department of Medicine.—J. A. E. Eyster, J. C. Flippin, J. H. Kastle.

University College of Medicine, Department of Medicine.—A. L. Gray, A. G. Brown, F. W. Howle.

University of Washington, School of Pharmacy.—Charles W. Johnson, Irvin W. Brandel, Albert H. Dewey; H. G. Byers, T. C. Frye, John Weinzirl.

Wisconsin Pharmaceutical Association.—Otto J. S. Boberg, Edward G. Raeuber, Ferd W. Thieman; Alternates Edward Williams, George Kestin, Richard Sommer.

University of Illinois, School of Pharmacy.—C. S. N. Hallberg, W. A. Puckner, W. B. Day; Alternates, A. H. Clark, C. M. Snow, E. N. Gathercoal.

Northwestern University, School of Pharmacy.—Oscar Oldberg, Harry Mann Gordin, Charles W. Patterson.

Indiana State Medical Association.—W. H. Foreman, Samuel Kennedy, James A. Mattison.

Purdue University, School of Pharmacy.—Harvey W. Wiley, Ernest G. Eberhardt, Charles E. Vanderkleed.

Valparaiso University, School of Pharmacy.—J. Newton Roe, George D. Timmons, A. F. Heineman; Alternates, Arthur Linton, John P. Sievers, Otis B. Nesbit.

University of Iowa, College of Medicine.—Charles S. Chase.

University of Iowa, College of Pharmacy.—Wilber J. Teeters.

Kansas Pharmaceutical Association.—Frank Holliday, W. S. Amos, Matt Noll; Alternate, S. J. Crumbine.

University of Kansas, School of Pharmacy.—L. D. Havenhill, E. H. S. Bailey, L. E. Sayre; Alternates, H. W. Emerson, F. W. Busheng, W. S. Dick.

Johns Hopkins University, Medical Department.—William S. Thayer, Thomas McCrae, Leonard G. Roundtree.

Massachusetts Medical Society.—Frank G. Wheatley, Charles H. Cook, Maurice P. V. Tyrode.

College of Physicians and Surgeons, Boston.—Ephriam Cutter.

University of Michigan, Department of Medicine and Surgery.—Charles W. Edmunds, Worth Hale, J. W. Trask.

Minnesota State Medical Association.—Ray Humiston; Alternate, F. J. Patton.

University of Minnesota, College of Pharmacy.—Frederick J. Wulling, W. A. Frost, Robert L. Morland.

New Jersey Medical Society.—Henry L. Coit, Alexander Marcy, Jr., Philip Marvel; Alternates, Isaac E. Leonard, Henry H. Davis, Joseph Tomlinson.

New Jersey Pharmaceutical Association.—George M. Beringer, Herman J. Lehmann, George H. White; Alternates, Edward B. Jones, Charles Holzhauer, H. H. Deakyne.

Albany Medical College.—Howard Van Rennselaer, Spencer Lyman Dawes, Victor Caryl Myers.

Long Island College Hospital.—Frank E. West, Elias H. Bartley.

University of Buffalo, Medical Department.—Eli H. Long, Dewitt H. Sherman, Edward J. Kiepe.

Syracuse University, College of Medicine.—William Dewey Alsliver, Frank P. Knowlton.

New York State Pharmaceutical Association.—Joseph Kahn, John Hurley, Joseph Weinstein.

Albany College of Pharmacy.—Willis G. Tucker, Alfred B. Huested, Theodore J. Bradley; Alternate, Harry B. Mason.

Brooklyn College of Pharmacy.—William C. Anderson, Henry W. Schimpf, A. P. Lohness; Alternates, Daniel C. Mangan. Tracy E. Clark, F. P. Tuthill.

University of Buffalo, Department of Pharmacy.—Willis G. Gregory, Frank E. Lock, John R. Gray; Alternates, George Reimann, Lee W. Miller, John C. Krieger.

---

THE Rochester Academy of Medicine held meetings during the month of February, 1910, as follows:

*General Medicine.*—Wednesday evening, February 2. Program: Immunity, Joseph Roby. Discussion was opened by J. R. Culkin.

*Surgery.*—Wednesday evening, February 9. Program: The systematic septic infections from tonsillar disease, John O. Roe.

*Obstetrics.*—Wednesday evening, February 16. Program: The vein sign in abdominal inflammations, William W. Skinner, Geneva, N. Y.

*Public Health.*—Wednesday evening, February 23. Program: Recent advances in our knowledge of the physiology of digestion, John R. Williams.

---

THE Buffalo Academy of Medicine held meetings during the month of February, 1910, as follows:

*A Stated Meeting* was held Tuesday Evening, February 1. Program: Surgery of the joints, J. B. Murphy, Chicago.

*Section on Medicine.*—Tuesday evening, February 8. Program: Gastric analysis in infants, DeWitt H. Sherman; Gastric hyperacidity, A. E. Woehnert.

*Section on Obstetrics and Gynecology.*—Tuesday evening, February 15. Program: Review in the treatment of ectopic pregnancy, J. Wesley Bovee, Gynecologist to Columbia Hospital for Women, George Washington Hospital and to the Government Hospital for the Insane, and Professor of Gynecology to George Washington University. Discussion was opened by C. C. Frederick.

*Section on Pathology.*—Friday evening, February 25. Program: Practical applications on the value of ammonia in the urine, by Professor J. B. Leathes, of England; The kidney in nephritis with illustrations, H. U. Williams.

---

THE Association of American Medical Colleges will hold its Twentieth Annual Meeting at the Hotel Belvedere, Baltimore, March 21 and 22, 1910.

